# Editorial: Insights in aquatic microbiology: 2023

**DOI:** 10.3389/fmicb.2024.1496983

**Published:** 2024-10-03

**Authors:** Jin Zhou, Michael Rappe

**Affiliations:** ^1^Shenzhen Public Platform for Screening and Application of Marine Microbial Resources, Institute for Ocean Engineering, Shenzhen International Graduate School, Tsinghua University, Shenzhen, China; ^2^Marine Biology, Hawai'i Institute of Marine Biology, SOEST, University of Hawai'i at Mānoa, Kāne'ohe, HI, United States

**Keywords:** microorganisms, marine, freshwater, aquatic microbiology, editorial

**Figure 1:**
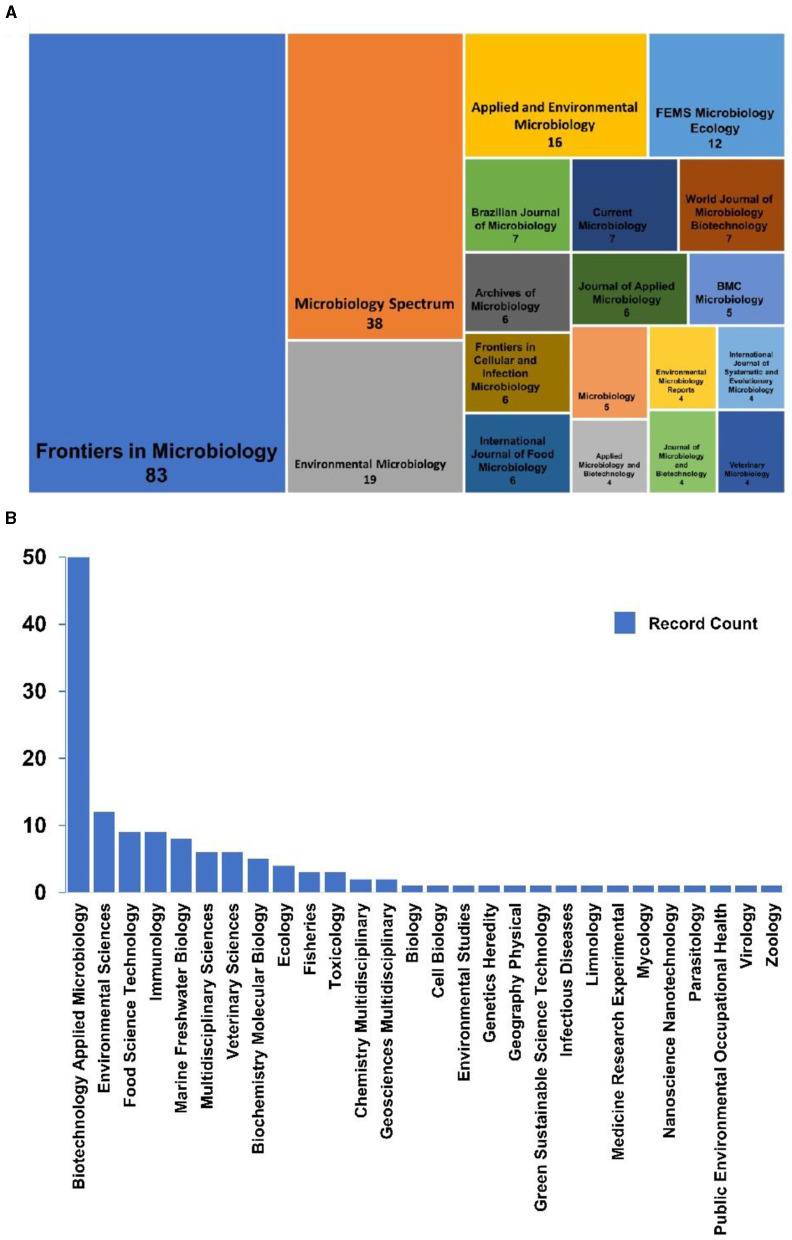
Titles and corresponding number of publications in journals that publish papers on the topic of Aquatic Microbiology during 2023 **(A)**. Information on the number of publications categorized under different topics within the search for Aquatic Microbiology **(B)**. These data have been obtained from the Web of Science.

Aquatic microbiology is a multifaceted and rapidly expanding field investigating the intricate interactions and complex dynamics of microorganisms within diverse aquatic ecosystems. This field covers a wide range of aquatic environments, from the vast expanses of the world's oceans to the serene depths of lakes, the winding currents of rivers, and the numerous other water bodies that sustain life on Earth. By studying these microbial communities, aquatic microbiologists seek to elucidate their roles in nutrient cycling, energy flow, and the overall health of the planet's aquatic systems. The section on Aquatic Microbiology, featured in the journal *Frontiers in Microbiology* and *Frontiers in Marine Science*, has emerged as a prominent publication in the field, providing leading research and insights into aquatic microbiology (Figure 1). A search on the Web of Science for the terms “aquatic” (all fields) AND “microbiology” (all fields) for the year 2023 identified 321 publications, with a notable 25.9% appearing in this specific section (Figure 1A). These publications covered several Web of Science categories, contributing to general microbiology, biotechnology, applied microbiology, and environmental sciences (Figure 1B). Aquatic microorganisms are a focal point of research, particularly in fields such as biogeochemical cycling, material metabolism, pollutant degradation, ecological restoration, and public health. Gaining a comprehensive understanding of their roles and behaviors in these contexts is essential for advancing scientific knowledge and effectively addressing environmental challenges.

In recent years, there have been significant new insights and discoveries in this field. The publications included in the current Research Topic, “*Insights in aquatic microbiology: 2023*,” cover a broad range of subjects that advance our understanding of this field. They address the following aspects in both fresh- and salt-water environment:

In freshwater ecosystem:

1. Bakenhus et al. (A domesticated photoautotrophic microbial community as a biofilm model system for analyzing the influence of plastic surfaces on invertebrate grazers in limnic environments) developed a standardized approach to investigate the impacts of plastic-associated biofilms on trophic interactions and biogeochemical cycles within three freshwater aquatic ecosystems. Utilizing this model system, we are now equipped to more profoundly comprehend how plastic pollution might alter the properties of biofilms, consequently affecting the health and functionality of aquatic ecosystems.
2. Peng et al. (Insight into diversity change, variability and co-occurrence patterns of phytoplankton assemblage in headwater streams: a study of the Xijiang River basin, South China) presented a comprehensive analysis of phytoplankton diversity in headwater streams across the Xijiang River basin, revealing a significant decrease in diversity with increasing altitude. The research highlights the “isolated island” effect of high altitudes on phytoplankton assemblages, characterized by reduced homogeneous selection and increased dispersal limitation. These findings are crucial for understanding the impact of environmental gradients on aquatic biodiversity and the potential consequences of climate change on these ecosystems.
3. Zhang et al. (Response of the microbial community structure to the environmental factors during the extreme flood season in Poyang Lake, the largest freshwater lake in China) investigated the response of microbial community structures to environmental factors during the extreme flood season in Poyang Lake. The experimental results demonstrated significant differences in bacterial communities between waterbody and sediment and revealed the abundance of genes related to human pathogens. This study contributes to understanding the health risks linked to flood events and supports the development of effective freshwater lake management and conservation strategies.

In saltwater ecosystem:

1. Lee et al. (Grazing impact of the calanoid copepods *Acartia* spp. on the toxic dinoflagellate *Alexandrium pseudogonyaulax* in the western coastal waters of Korea) found that the ingestion rates of the copepod *Acartia* spp. on the harmful algae *Alexandrium pseudogonyaulax* increased with higher prey concentrations, suggesting that copepods may play a role in mitigating algal blooms. The findings illustrate the ecological significance of copepods in controlling toxic dinoflagellates and enhance our understanding of marine planktonic food webs.
2. Rey Redondo et al. (Genomic characterization and ecological distribution of *Mantoniella tinhauana*: a novel Mamiellophycean green alga from the Western Pacific) identified a novel marine alga, *Mantoniella tinhauana*, in the Western Pacific, marking a significant advancement in marine biology. Their genomic sequencing and analysis solidify the alga's status as a distinct species and elucidate its global distribution. This study enhances our understanding of marine microbial diversity and distribution while offering new molecular insights into their roles in marine ecosystems.
3. Shan et al. (An abrupt regime shift of bacterioplankton community from weak to strong thermal pollution in a subtropical bay) revealed a dramatic shift in the bacterioplankton community structure within a subtropical bay, triggered by thermal pollution from a nuclear power plant. These findings indicate a critical ecological transition and suggest that we need to monitor and mitigate the environmental impacts of industrial activities, especially in the context of rising global temperatures.
4. Ugarelli et al. (Microbiomes of *Thalassia testudinum* throughout the Atlantic Ocean, Caribbean Sea, and Gulf of Mexico are influenced by site and region while maintaining a core microbiome) revealed a resilient core microbiome within *Thalassia testudinum*. Despite vast geographical spans, the seagrass maintains a stable microbial community structure, shaped by local environmental factors. These findings hold significant implications for the conservation and management of seagrass beds, particularly in the context of global change, offering us new tools for monitoring and preserving the health of these critical ecosystems.
5. Walker et al. (Above and below-ground bacterial communities shift in seagrass beds with warmer temperatures) investigated the impact of warmer temperatures on seagrass and its associated microbial communities in Lake Macquarie, Australia. The research examined the potential for temperature-induced changes in seagrass ecosystems, emphasizing the importance of understanding microbial responses to climate change for the conservation of these vital habitats. It provides critical insights into how climate change, particularly ocean warming, could alter the delicate balance of seagrass ecosystems.
6. Yang et al. (Unraveling the important role of comammox Nitrospira to nitrification in the coastal aquaculture system) highlighted the critical role of comammox Nitrospira in nitrification processes within coastal aquaculture systems. Their comprehensive assessment of nitrifying communities in fish ponds with different feeding levels deepens our knowledge of microbial dynamics in aquaculture and offers strategic insights for sustainable nitrogen management and water quality control.

These publications in this section provide new insights into the stability and resilience of microbial ecosystems in aquatic systems, water quality management, nitrogen cycling, and the protection and restoration of ecosystems, thereby advancing the field of aquatic microbiology. They enhance our understanding of microbial community dynamics and highlight the critical role of microorganisms in maintaining the health and functionality of fresh- or salt-water environments. As research in aquatic microbiology progresses rapidly, it is crucial to adopt new technologies to improve research capabilities. Developing and utilizing technologies such as artificial intelligence and machine learning will facilitate the analysis of complex ecological data, allowing for more precise identification of changes in microbial communities and their impacts on water quality, ultimately leading to a deeper understanding and enhanced predictive capabilities. Additionally, fostering innovative scientific thinking and interdisciplinary collaboration is important. By encouraging novel research approaches and focusing on real-world problems, we can move beyond traditional studies. Strengthening collaboration among disciplines such as ecology, microbiology, hydrology, and environmental science will provide a more comprehensive analysis and resolution of issues within aquatic ecosystems. Finally, developing science-based management policies and promoting public education are essential. Updating water quality management policies and ecological protection measures based on the latest research ensures they are grounded in scientific evidence. Moreover, increasing public awareness about the importance of microorganisms in maintaining aquatic health will foster greater public support and involvement in water protection efforts. Through these strategies, we can better understand and manage the role of aquatic microbiology in ecosystem dynamics, optimize water quality management, and promote the sustainable development of ecosystems.

